# Real world outcomes of biopsy‐proven oncocytic neoplasm of the kidney managed by surveillance

**DOI:** 10.1002/bco2.141

**Published:** 2022-03-03

**Authors:** Flora E. Rodger, Keiran Brown, Steve Leung, Jack Coode‐Bate, James Armitage, Anne Warren, Jane Hendry, Grant D. Stewart, Alex Laird, Grenville M. Oades

**Affiliations:** ^1^ Department of Urology Queen Elizabeth University Hospital Glasgow UK; ^2^ Department of Urology Western General Hospital Edinburgh UK; ^3^ Department of Urology The University of Edinburgh, Western General Hospital Edinburgh UK; ^4^ Department of Urology University Hospitals Plymouth NHS Trust Plymouth UK; ^5^ Department of Urology Addenbrookes Hospital, Cambridge University Hospitals NHS Trust Cambridge UK; ^6^ Department of Pathology Addenbrookes Hospital, Cambridge University Hospitals NHS Trust Cambridge UK; ^7^ Department of Urology University of Cambridge Cambridge UK

**Keywords:** biopsy, chromophobe carcinoma, oncocytic, oncocytoma, surveillance

## Abstract

**Objectives:**

To evaluate outcomes of patients diagnosed with oncocytic renal neoplasms on routine renal mass biopsy and to describe the natural history of these tumours when managed with surveillance as opposed to immediate intervention. To report disease‐specific survival.

**Patients and methods:**

Patients were identified from a retrospective review of pathology databases from three tertiary referral centres that utilise renal mass biopsy in routine clinical practice. All patients with biopsy‐proven oncocytic tumours were included and a retrospective review of online patient records was undertaken.

**Results:**

There were 184 biopsy‐proven oncocytic renal neoplasms identified in 172 patients. There were two biopsy complications (both pneumothorax, Clavien–Dindo Grade I). Of these lesions, 135 were reported as oncocytomas or oncocytic renal neoplasms that were not further classified and 37 were reported as chromophobe carcinoma (ChRCC). The median age at diagnosis was 70 (33–88). The average tumour diameter at diagnosis was 33 mm. One hundred seven tumours were initially managed with surveillance (including 13 ChRCC) with a minimum follow‐up of 6 months and a median of 39 months (6–144) whereas 49 patients underwent immediate treatment. The mean growth rate across all oncocytic renal neoplasms managed by surveillance was 3 mm/year. There was no statistically significant difference in growth rates between oncocytic renal neoplasms and ChRCC. Thirteen patients with oncocytic renal neoplasms initially managed by surveillance moved on to an active management strategy during follow‐up. The clinical indication given for a change from surveillance was tumour growth in 12 cases and patient choice in 1 case. Where definitive pathology was available, there was 85% concordance with the biopsy. There were no cases of development of metastatic disease or disease‐related morbidity or mortality during the study.

**Conclusions:**

This multicentre retrospective cohort study supports the hypothesis that selected biopsy‐proven oncocytic renal neoplasms can be safely managed with surveillance in the medium term. Routine renal mass biopsy may reduce surgery for benign or indolent renal tumours and the potential associated morbidity for these patients.

## 1 INTRODUCTION

The term oncocytic renal neoplasms describes a spectrum of renal tumours with oncocytic features ranging from benign Oncocytomas to malignant tumours such as chromophobe carcinoma (ChRCC). The 2016 WHO Classification^1^ recognises oncocytoma and ChRCC as distinct entities; however, with advances in immunohistochemical and molecular testing, diagnosis is becoming more nuanced with several other tumours with oncocytic features now described. More recently, it has been appreciated that many of the low‐grade‐appearing oncocytic tumours show indolent behaviour and the term ‘oncocytic renal neoplasm of low malignant potential, not further classified’ is now commonly used when it is difficult to categorise such tumours.^2^ An oncocytic renal neoplasm diagnosed on renal biopsy can pose a significant challenge to the histopathologist and the clinician with a real risk of overtreatment of indolent disease.

The diagnosis of renal tumours has increased in the last decades with much of this increase being put down to the expanding use of cross‐sectional imaging.^3^ It is recognised that a significant number of renal masses are benign with almost 50% of tumours less than 1 cm falling into this category. However, the likelihood of malignancy increases with larger masses, and only 6.3% of tumours that are greater than 7 cm or higher have been shown to be benign.^4^ Despite this, overtreatment of benign tumours appears common be with 30% of tumours removed by partial nephrectomy in the United States having non‐malignant histology.^5^ In the United Kingdom, the reported figure is lower with 18% of partial nephrectomies performed for benign disease.^6^

Routine renal mass biopsy may reduce surgery for benign tumours and the potential for short‐term and long‐term morbidity associated with these procedures.^7^ However, the routine use of preoperative biopsy is not recommended by the American Urological Association or the European Association of Urology (EAU). The underutilisation of this technique is due to several concerns including difficulties in distinguishi

ng between different types of benign and malignant oncocytic neoplasms, the safety of core biopsy and the accuracy of grading on such samples. Significant nondiagnostic rates have also been reported,^8^ and concerns have been raised about a low negative predictive value suggesting that a non‐malignant result may not be truly representative.

Oncocytoma is the second most common benign renal tumour accounting for 3%–7% of all renal masses whilst ChRCC is the third commonest subtype of renal malignancy making up about 5% of all renal cancers.^1^ If the routine use of renal mass biopsy is to reduce the morbidity associated with overtreatment of benign renal tumours with surgery or ablation, there has to be an expectation that oncocytomas, and other oncocytic renal neoplasms can be properly classified and safely managed by surveillance or indeed discharged with a definitive diagnosis.

Previous studies have reported on the growth kinetics of oncocytic renal neoplasms; however, they are often limited by small numbers and short follow‐up. In this study, we will report data on a large series of oncocytic renal neoplasms from three centres where routine renal mass biopsy has been adopted as standard of care. We aim to show how renal mass biopsy is safe to use in a contemporary clinical setting. We report the outcomes of patients diagnosed with oncocytic renal neoplasms when managed with surveillance or immediate intervention and describe the natural history of tumours managed with surveillance.

## 2 PATIENTS AND METHODS

In this multicentre retrospective cohort review, patients with a diagnosis of an oncocytic renal neoplasm on a renal mass biopsy were identified from pathology records of three tertiary referral centres that have used routine small renal mass biopsy in their practice from 2014 onwards: The Queen Elizabeth University Hospital, Glasgow; Western General Hospital, Edinburgh; and Addenbrooke's Hospital, Cambridge University Hospital's NHS Foundation Trust. All lesions <4 cm were considered for biopsy as well as selected larger lesions where it was considered biopsy may influence management decisions.

The primary outcome measure was tumour growth rate calculated by measuring maximum tumour diameter at first and last scan and time of follow‐up. Initial and final management decisions, including the reason for any change of management, and the clinical outcome were recorded. Demographic data were recorded as well as tumour complexity (PADUA score) and multifocality, symptomatic status, estimated glomerular filtration rate (eGFR) and biopsy complications. The surveillance protocol and imaging modality were at the discretion of the treating physician. Tumours where less than 6 months of follow‐up was available were excluded from the analysis.

Biopsies were undertaken according to local protocol. They were performed under local anaesthetic and either computed tomography (CT)‐ or ultrasound scan (USS)‐guided using a 16 or 18G needle and a coaxial technique. Two or more cores were sampled.

The diagnosis and classification of oncocytic renal neoplasm was made by specialist histopathologists after analysis of the biopsy using the classically described criteria of granular cytoplasm following staining with haematoxylin and eosin. Immunohistochemistry included staining for CD117, PAX‐8, CK7, CD15, vimentin, CD10 and RCC was also employed. Classification was made with reference to the WHO Classification of Tumours of the Urinary System. For the purposes of analysis, oncocytic neoplasms were grouped based on histopathology reports into those favouring benign or indolent pathology (where histopathologists reported oncocytoma, oncocytic renal neoplasms favouring oncocytoma or oncocytic renal neoplasms where no other classification was given) and those where the histopathology report favoured a diagnosis of ChRCC.

Statistical analysis was performed in SPSS (v27, IBM Chicago) using one‐way ANOVA for continuous variables and *χ* ^2^ testing for categorical variables with significance taken at *p* < 0.05.

## 3 RESULTS

The total cohort of oncocytic renal neoplasms included 184 lesions in 172 patients. The baseline demographic, clinical and tumour data are depicted in Table 1. The majority of patients were male with a median age at diagnosis of 69 years and a range of 33–88 years. The median tumour size at diagnosis was 33 mm. The majority of tumours were less than 4 cm; however, there were 40 that measured between 4 and 7 cm and 6 greater than 7 cm. Of patients, 81% were asymptomatic at presentation. Ten patients had a known genetic predisposition at diagnosis. In eight cases, this was Birt‐Hogg‐Dube Syndrome, and in the remaining two cases, there was a known mutation in the *SDHB* gene.

**TABLE 1:** Baseline demographic data and tumour characteristics

			Oncocytoma/indolent	ChRCC
Total patients	*N*	172	135	37
Male	%	71	77	73
Age	Median (range)	70 (33–88)	69 (49–88)	68 (33–79)
eGFR <60 mL/min at diagnosis	%	19	21	16
Symptomatic at presentation	%	19	18	22
Total lesions	*N*	184	147	37
Size at diagnosis	Average (mm)	33 (10–89)	33 (10–89)	33 (11–75)
PADUA score	Median (range)	8 (6–13)	8 (6–13)	8 (7–12)
Tumour <4 cm	*N*	138	112	26
Tumour 4–7 cm	*N*	40	30	10
Tumour >7 cm	*N*	6	5	1

In 102 tumours (55%) specialist histopathologists reported Oncocytoma or an oncocytic neoplasm favouring Oncocytoma. There were 34 cases (18%) where the histopathologist reported ChRCC or an oncocytic lesion favouring ChRCC. Three cases were reported as hybrid oncocytic/chromophobe tumours, and in the remaining 45 lesions (24%), the histopathologist did not further subclassify the oncocytic neoplasm (this group was considered as indolent, oncocytic renal neoplasms of low malignant potential for the purposes of this study and grouped with the Oncocytomas). In six cases, a second renal mass biopsy was performed. This was because the initial biopsy was nondiagnostic in one case and in five cases, a repeat biopsies was undertaken due to growth of the tumour. In four of these cases, an initial diagnosis of oncocytoma was confirmed and in the remaining case an oncocytic tumour that was not further classified remained uncategorised and subsequently underwent a radical nephrectomy. There were two reported biopsy complications (both pneumothoraces, Clavien–Dindo Grade I).

**FIGURE 1:**
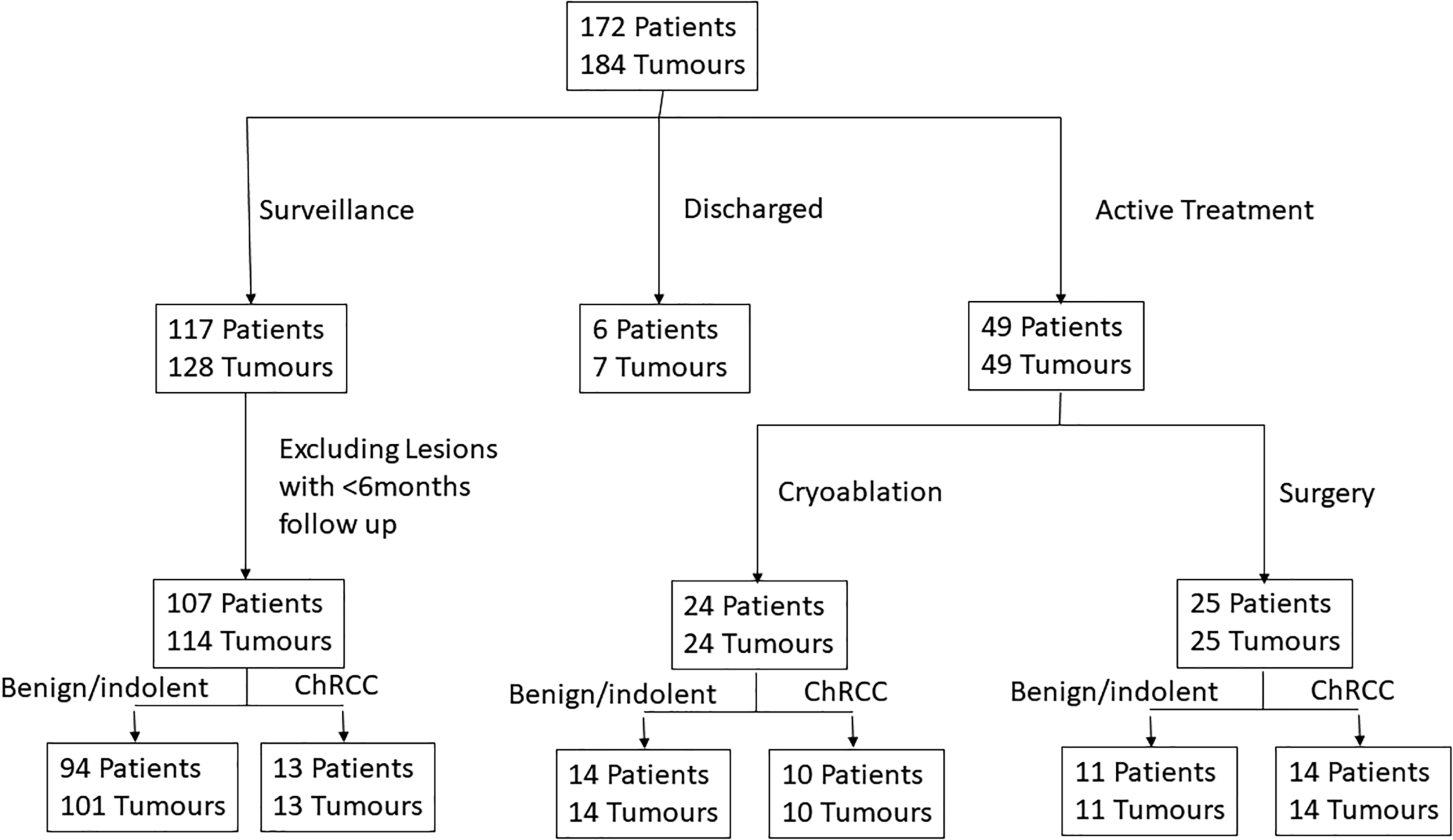
Consort diagram of patients and tumours

Forty‐nine patients (28%) elected to undergo immediate treatment of at least 1 tumour whilst 117 (68%) patients were initially managed with surveillance. Of these patients, 107 (62%) had at least 6‐months follow‐up and were included in the analysis. Six (3%) were discharged after diagnosis. Details are given in the consort diagram (Figure 1). Of the patients undergoing immediate treatment, 24 had cryoablation and 25 had surgery (20 [80%] of these had a partial nephrectomy and 5 [20%] radical nephrectomy). Where the histopathologist had subcategorised the oncocytic renal neoplasm on the core biopsy, there was concordance with the definitive histopathology report following surgery in 85% of cases. In four (15%) cases initially classified as oncocytoma, there was discordance with final pathology results where the definitive histopathology was reported as follows: ChRCC (*n* = 2), oncocytic variant of papillary RCC (*n* = 1) and oncocytic RCC unclassified (*n* = 1). Importantly, there was no disease‐related morbidity or mortality in patients undergoing immediate treatment throughout the follow‐up period.

**FIGURE 2:**
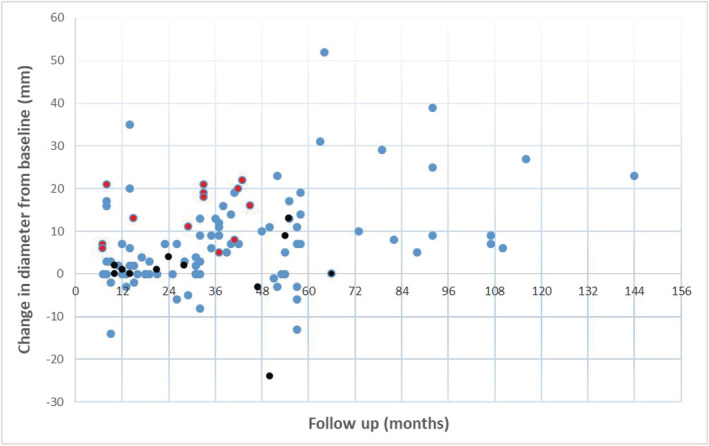
Change in diameter from baseline of oncocytic renal neoplasms undergoing surveillance. Points in red show cases where policy of surveillance changed. Points in black represent tumour where initial pathology favoured chromophobe carcinoma (ChRCC)

One hundred fourteen tumours in 107 patients (62%) were initially managed with surveillance and had at least 6‐months follow‐up. The surveillance protocol was at the discretion of the treating physician. The maximum diameter of the tumour was measured on the initial and final imaging. The median diameter of tumours initially managed by surveillance was 33 mm (11–89), but this included 29 (25%) of tumours >4 cm. There was a median follow‐up of 39 months (6–144) including 52 (46%) of tumours followed up for greater than 3 years and 15 (13%) for greater than 5 years. The mean growth rate across all oncocytic renal neoplasms was 3.0 mm/year, but there was a wide range within the surveillance cohort with 13 lesions actually regressing in size and a minimum growth rate of −18.7 mm/year to a maximum of 31.5 mm/year. There was no statistical difference in average growth rates for tumours less than 4 cm when compared with larger tumours. (2.8 mm/year vs. 3.1 mm/year, *p* = 0.88). Importantly, there was no statistical difference in growth rate between oncocytic neoplasms favouring benign or indolent pathology and those diagnosed as ChRCC (3.3 mm/year vs. 0.4 mm/year, *p* = 0.67), although the number of lesions where the renal mass biopsy favoured a malignant diagnosis but were initially managed with surveillance was small (13 lesions) (Figure 2).

Of patients, 19% had an eGFR of <60 mL at diagnosis, 27 (24%) in the surveillance group and 7 (14%) in the treatment group. Follow‐up data on renal function were available in 107 (69%) patients, 62 (58%) in the surveillance group and 42 (86%) in the treatment group. In the surveillance group, there was an average decrease in eGFR of 0.9 mL/min/year with a median follow‐up of 55 months compared with the immediate treatment group where there was an average decrease in eGFR of 0.4 mL/min/year with a median follow‐up of 54 months (*p* = 0.01).

Thirteen patients with benign or indolent pathology initially managed with surveillance were moved over to an active management strategy during the follow‐up period. Five of these patients were managed surgically, and eight patients underwent ablation. The clinical indication given for a change from a surveillance strategy was tumour growth in 12 cases (These lesions grew on average 7.5 mm/year) (Figure 2) and patient choice in one case. In the small group of ChRCC patients initially managed with surveillance, a single patient subsequently elected to have their tumour ablated and a further patient went on to have a radical nephrectomy after commencing renal replacement therapy where a diagnosis of ChRCC was confirmed. In no case was symptomatic progression recorded as a reason to abandon a surveillance strategy. Where definitive pathology was available following surgical treatment, there was 100% concordance with the initial core biopsy.

Eleven deaths (10%) were recorded in patients undergoing surveillance. All deaths were from causes unrelated to the diagnosis of oncocytic renal neoplasm. In five cases, cause of death was recorded as pneumonia, in three cases ischaemic heart disease, one patient died of unrelated malignancy, one of advanced Parkinson's disease and one patient died following a hip fracture. Of note, there were no cases of development of metastatic disease or disease‐related morbidity or mortality during the study in any of the surveyed patients.

When comparing differences between patient cohorts selected by the treating clinician that underwent immediate treatment or initial surveillance, only age and the histopathology of a biopsy favouring ChRCC were found to be significant. Patients undergoing immediate treatment were likely to be younger at 62.3 years, compared with 71.1 years (*F* = 17.3, *p* < 0.01) and have an initial biopsy favouring ChRCC (47% vs. 13%, *X* ^2^ = 2.4, *p* < 0.01). When looking at sex, genetic predisposition, multifocality, initial size of lesion, decreased eGFR at diagnosis and nephrometry scores, there were no statistically significant differences between surveillance and immediate treatment groups (*p* = 0.39–0.92.) (Table 2).

**TABLE 2:** Disease and patient factors in patients managed with surveillance and those immediately treated

	Surveillance (*N* = 107)	Treatment (*N* = 49)	*p* value
Sex	71%, 29% F	67%M: 33%F	0.98
Age	71.1 (*SD* 8.3)	62.3 (*SD* 11.0)	<0.01 *
Biopsy pathology favouring ChRCC	14 (13%)	23 (47%)	<0.01*
PADUA score	8.04 (*SD* 1.7)	8.47 (2.1)	0.30
Genetic predisposition (%)	6 (5.6%)	5 (10.2%)	0.35
Multifocal lesions (%)	37(34.9%)	11(22.4%)	0.40
Initial size (mm)	32.7 (*SD* 15.2)	33.5 (*SD* 15.0)	0.78
eGFR at diagnosis	54.2 (*SD* 26.6)	44.4 (*SD* 8.3)	0.64

*Note*: Mean values and standard deviation (*SD*) with significant differences highlighted (*).

Abbreviation: eGFR, estimated glomerular filtration rate.

## 4 DISCUSSION

With the increasing use of abdominal ultrasound and cross‐sectional imaging, there has been a surge in the detection of renal tumours.^7^ Although surgical treatment remains the standard of care in many centres for larger renal tumours, it is recognised that a significant proportion, particularly of smaller tumours, may be benign and there is a concern that overtreatment of these tumours may result in unnecessary short‐ and long‐term morbidity for some of these patients.^8^ Treatment of such lesions without a prior histological diagnosis is becoming increasingly contentious because of this. The EAU currently recommends that renal mass biopsy is performed prior to ablative therapy and in patients where surveillance is being considered as a management strategy but does not recommend routine biopsy.

In this current study, we show that in a contemporary UK clinical setting, when routine biopsy is employed for all small renal masses, selected oncocytic renal neoplasms can be safely managed in the medium term with a surveillance strategy without development of disease related morbidity or mortality. One hundred fourteen tumours in 107 patients (62%) were initially managed with surveillance in this study and were followed up for a median of 39 months without the development of metastases or any disease‐related morbidity. Growth rates reported in previous studies have ranged from 1.4–5.2 mm/year^9^, ^10^, ^11^, ^12^, ^13^, ^14^ that is in keeping with our reported rate of 3 mm/year although this was extremely variable, and it should be noted that some patients with ChRCC managed on a surveillance protocol actually showed shrinkage of their tumour.

The EAU guidelines recommend nephron sparing surgery is offered to all patients with T1 tumours. In the United Kingdom, 3.7% of all renal surgical procedures are performed for oncocytomas, and few of these patients had preoperative biopsies. We would argue that is becoming increasingly difficult to justify surgical treatment for benign or indolent tumours as the default option, particularly in a comorbid population and considering 20% of the patients are known to have in hospital complications and 4% have complications of Clavien–Dindo Grade III or above with a mortality rate of 0.1%.^6^ Indeed, routine renal tumour biopsy has been shown to reduce surgery for benign tumours and the potential for short‐term and long‐term morbidity associated with these procedures by two thirds.^8^

Perhaps the biggest concern for clinicians not using routine renal mass biopsy in their practice is the ability to distinguish between types of oncocytic renal neoplasms, especially oncocytoma and ChRCC, and accurately grade a tumour. There is a fear that some lesions may be inappropriately labelled as benign or indolent. Oncocytic renal tumours present a challenge for the histopathologist. The 2016 WHO classification of tumours of the urinary system recognises ChRCC and oncocytoma as separate entities, both of which can display oncocytic features and obviously have different clinical significance. In our study, definitive histopathology was available in 31 cases following surgery and the pathology of lesions reported on an initial renal biopsy showed concordance with the definitive pathology in 87% of cases. This is better than a recent meta‐analysis reporting on over 200 renal mass biopsies of oncocytic renal neoplasms suggestive of oncocytoma and giving a positive predictive value (PPV) of 67% compared with final pathology post‐surgery, with significant heterogeneity noted between the studies.^15^ It is possible that adopting a surveillance strategy for all oncocytic renal tumours may result in a small number of patients with ChRCC or other malignant oncocytic renal neoplasms being erroneously placed in a surveillance programme. It is important to point out however that patients with ChRCC have a low risk of tumour progression, metastasis and cancer‐specific death. The 10 year cancer‐specific survival for T1a ChRCC approaches 100%.^16^ Probably the most striking finding of the current study is that no patients with oncocytic renal neoplasms developed disease‐related morbidity or mortality in this study despite a median follow‐up of over 3 years. This is in contrast to comparable series of small renal mass surveillance without a biopsy where a small but significant 2% of patients have been shown to progress to metastatic disease, presumably due to the high incidence of malignant tumours included.^17^

The safety of renal mass biopsy has been questioned; however, in our series, there were no significant biopsy complications. A previous meta‐analyses has shown percutaneous renal tumour biopsy to be safe with major complications in <0.1% of cases and transfusion rates of 0.1%. Seeding has also historically been a concern; however, this is thought to be rare.^18^ Questions have also been raised about the diagnostic accuracy of renal mass biopsy; however, the same meta‐analysis reported sensitivity and specificity of diagnostic renal mass core biopsies of 99.1% and 99.7%, respectively.

Some authors have expressed concern that tumour growth, even of non‐malignant lesions, will lead to a decline in renal function and development of symptoms. In one recent study the authors concluded that surveillance was associated with a greater decline in renal function than partial nephrectomy in patients with Oncocytoma and so justifying surgery for some of these tumours.^19^ However, a large recent series has concluded that renal function does not seem to be negatively impacted by growing oncocytomas.^20^ Our data show a small but significant decrease in renal function of less than 1 mL/min/year of follow‐up in the surveyed patients when compared with patients undergoing immediate treatment. This difference could potentially be explained by the larger number of patients with chronic kidney disease at baseline in this group (24% compared with 14%), but long‐term monitoring of renal function in patients with oncocytic renal tumours managed with surveillance and in future studies of the management of oncocytic renal neoplasms would seem appropriate.

We are aware of a few similar series looking at surveillance of oncocytomas; however, this study is one of the largest cohorts in the literature with one of the longest follow‐up periods. Another strength of the current study is that it includes all oncocytic renal neoplasms and not just oncocytomas making in more relevant to a contemporary clinical setting. A criticism of our study could be that it specifically looked at a cohort of patients selected for surveillance by the treating physician and not all oncocytic neoplasms therefore possibly introducing bias. As discussed, younger patients with pathology favouring malignancy were more likely to be immediately treated. However, what it does show is that the real world selection of patients for surveillance, although not well defined, appears to be effective at identifying patients where this is a safe strategy. This study does highlight an inconsistency in our current approach to decision making around the management of small renal masses. In this cohort, patients were more likely to be managed with ablation or surgery if they were diagnosed with an oncocytic renal neoplasm at a younger age; however, we know the incidence of benign tumours is higher in younger age groups.^21^

Enhanced immunohistochemical and molecular testing is now increasing our ability to differentiate between oncocytic renal neoplasms and has also lead to the identification of several other distinct entities with oncocytic features such as eosinophilic solid and cystic renal cell carcinoma (ESC RCC), low‐grade oncocytic renal tumour (LOT) and high‐grade oncocytic renal tumour (HOT). Modern imaging techniques such as diffusion‐weighted magnetic resonance imaging (MRI) and technetium‐99m ([99mTc])‐sestamibi single‐photon emission computed tomography/x‐ray CT (SPECT/CT) may also have a role in differentiating between oncocytomas and malignant tumours.^22^, ^23^ The existence of a multitude of oncocytic tumours and the ability to diagnose them on routine renal mass biopsy and MRI will be important in the future to allow us to personalise treatment for individual patients.

If adopting a surveillance strategy for oncocytic renal tumours is to become standard further work is needed to develop a protocol. In most instances clinicians rely on changes in diameter of the lesion on cross‐sectional imaging or ultrasound to influence management decisions. Trigger points for definitive intervention following an initial period of surveillance have not been clearly identified although a fast growth rate was the most commonly cited reason here. Previous reports have suggested that oncocytomas measuring more than 5 cm or growing more than 5 mm/year should be definitively treated.^12^ These arbitrary cut offs are not supported by our data as we have shown that oncocytic renal neoplasms grow at widely varying rates with no difference between tumours identified as benign, indolent or malignant. Indeed, some ChRCC in this series actually regressed over time.

In conclusion, we hope that this paper challenges a perceived wisdom that an enhancing mass in the kidney represents a surgical lesion that automatically requires excision without the need for a pretreatment biopsy. We acknowledge that the accurate diagnosis of oncocytic renal neoplasms relies on coordinated multidisciplinary expertise in urology, interventional radiology and histopathology. Where such resources exist, selected patients with oncocytic renal neoplasms diagnosed on renal mass biopsy can be safely managed conservatively with surveillance without the development of disease‐related morbidity or mortality in the medium term. Advances in immunohistochemistry, genetics and imaging in the future will allow more accurate categorisation of these tumours enabling a more individualised approach to management.

## DISCLOSURE OF INTERESTS

None.
